# Weisun Tao: a pioneer of biochemistry in China

**DOI:** 10.1007/s13238-017-0383-9

**Published:** 2017-03-07

**Authors:** Tianwei He

**Affiliations:** School of Life Sciences, Jilin University, Changchun, 130012 China

**Figure 1:**
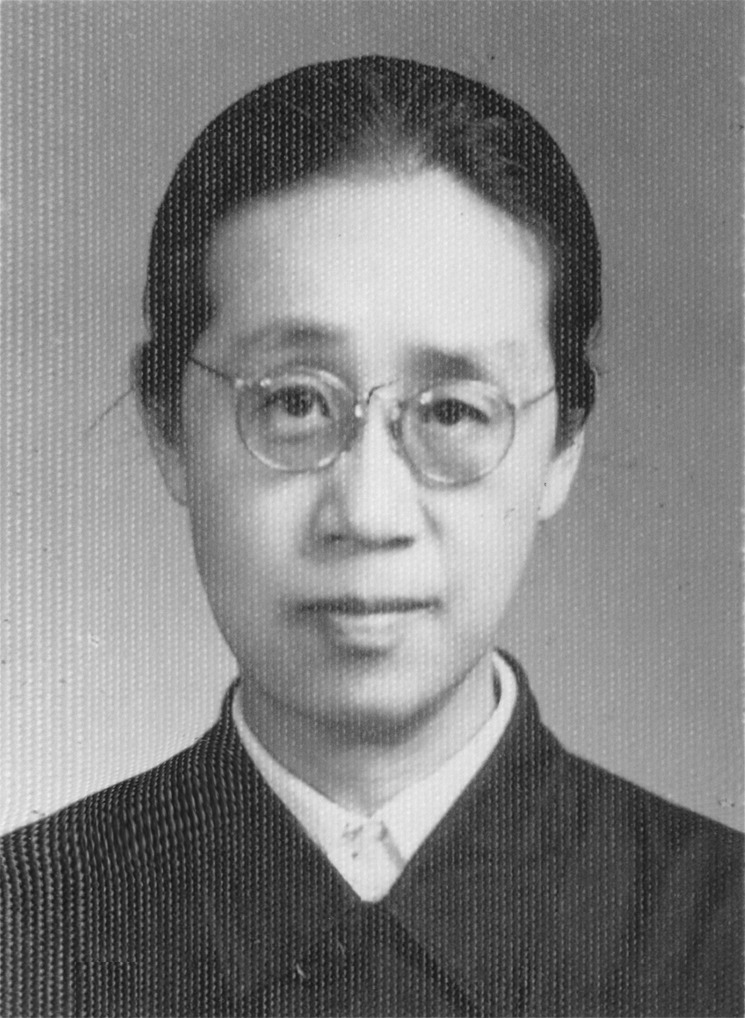
Professor Weisun Tao (Feb. 20, 1895–Dec. 11, 1982)

Professor Weisun Tao is a distinguished educator, a pioneering scientist of biochemical researches and one of the founders of the protein chemistry research in China (Fig. 1). She is a main founder of the Department of Chemistry at Utopia University, the Department of Chemistry and the Department of Biology at Jilin University. She was appointed as a professor in Utopia University, Northeast Institute of Technology, Jilin University, and the honorary director of the Chinese Biochemical Society. She also established the disciplines of organic chemistry and biochemistry at Jilin University.

Prof. Tao was born in 1895 in Wuxi, Jiangsu province. In 1902, she attended elementary school in Shanghai. She went to Japan with her father in 1906, who was then studying in Meiji Law School (now Meiji University). In 1914, after finishing her elementary and middle school studies, Prof. Tao was enrolled in Tokyo Women’s Higher Normal School. After graduation, she came back to China in 1918, and started teaching chemistry in Beijing Women’s Higher Normal School. In 1919, Prof. Tao went to America for further education. She obtained Master’s Degree of Science from Columbia University in 1921 and Master’s Degree of Education from Cornell University in 1923 (Tao, 2003). During that time, Prof. Tao has visited and studied at multiple famous chemical research institutions in the United Kingdom, Germany, France, Belgium, the Netherlands, and Switzerland. In the winter of 1923, she came back to China and was appointed as a professor in chemistry at Utopia University in Shanghai. In September 1927, Prof. Tao decided to go to Japan again for the doctoral study under the guidance of Shigeru Komatsu, a famous organic chemist in the College of Science, Kyoto Imperial University. There, Prof. Tao carried out a systematic study on the chemical change in the starch storage before and after rice germination, sugars change in rice germination at different temperatures, and hydrolysis of starch by diastase at different temperatures. In the autumn of 1931, Prof. Tao went back to China and continued to work as a professor of chemistry in Utopia University. In July 1932, Prof. Tao completed her doctoral dissertation entitled *Biochemical Studies on Rice Starch* and received her Doctoral Degree of Science (Komatsu, 1996). She was the first Chinese woman who obtained a doctoral degree in Japan. In the same year, she founded and served as the head of the Department of Chemistry. During that time, Prof. Tao took education as her priority and taught more than ten courses. Besides teaching, she also conducted researches on chemical components of ripe Huangyan orange and the essential oil in the ripe Fu orange fruit. From 1935 to 1944, Prof. Tao also held the position as a researcher in the Shanghai Science Institute. In 1940, Prof. Tao developed the first batch of glucose that can be used for injection in China. In the 1940s, Prof. Tao participated in the establishment of Shanghai Datong Chemical Industry Factory and Shanghai Yixin Chemical Manufacturing Factory, which was a pioneering contribution to chemical reagent industry in China.

**Figure 2:**
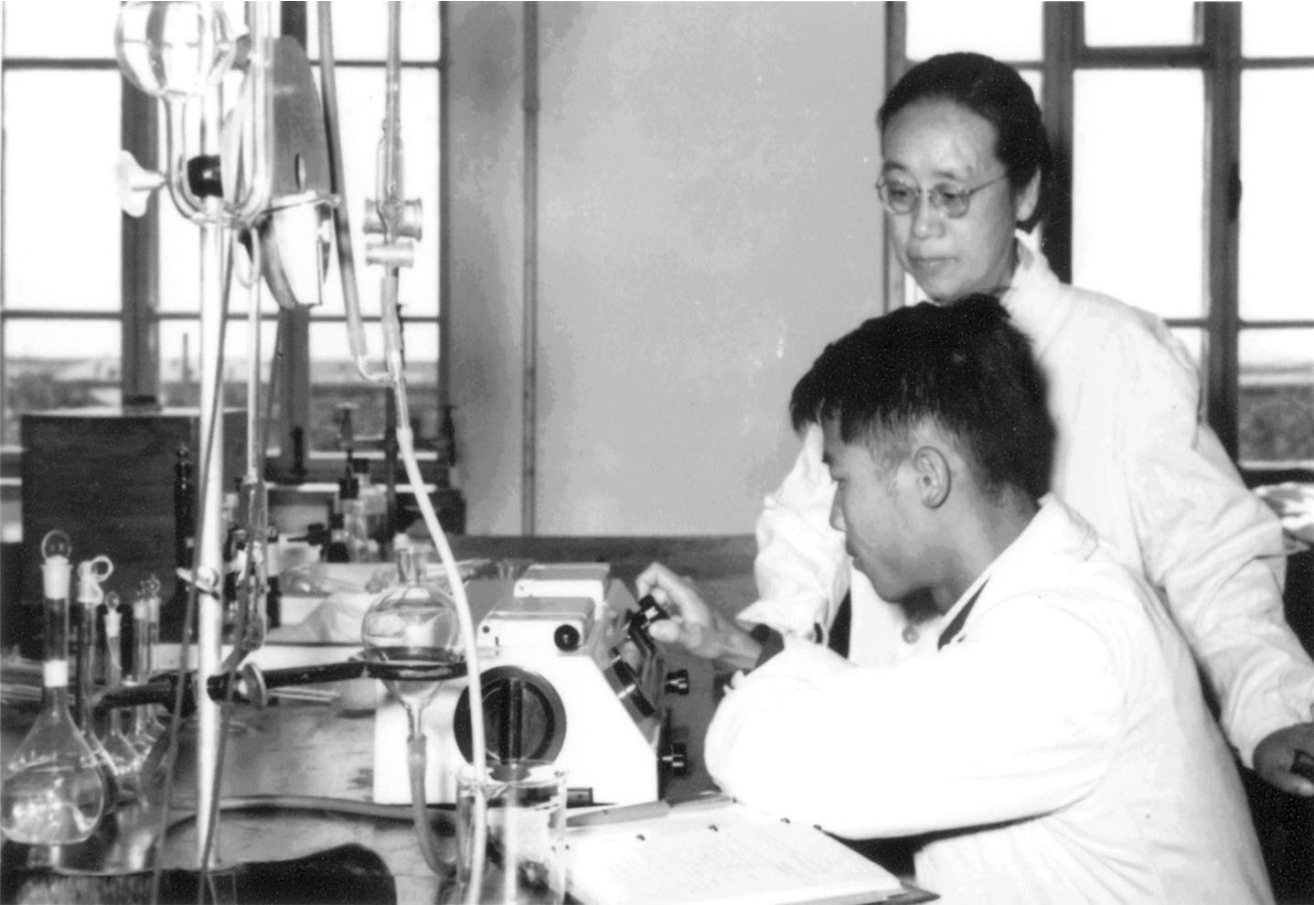
Professor Weisun Tao was guiding student in the laboratory

In September 1950, shortly after the founding of People’s Republic of China, Prof. Tao gave up her comfortable life in Shanghai and went to Shenyang to join in the Department of Chemical Engineering in Northeast Institute of Technology as a professor in response to the Communist Party’s encouragements to support the development of education and science in the northeastern area. In October 1952, in accordance with the arrangements of the Ministry of Education, Prof. Tao went to Changchun and founded the Department of Chemistry in the Northeast People’s University together with Profs. Liusheng Tsai, Auchin Tang, and Shizhi Guan. Among the four majors available in the Department of Chemistry: inorganic chemistry, organic chemistry, analytical chemistry, and physical chemistry, Prof. Tao was mainly responsible for the establishment of organic chemistry major (Fig. 2). At the beginning, it was very difficult. There were no laboratories, experimental instruments and reagents. Researchers have to conduct experiments in the basement: they used wooden boards to build the benches and used inkbottles as alcohol lamps. With all those efforts, students were finally able to do experiments. In August 1958, Northeast People’s University was renamed as Jilin University. In June 1960, Prof. Tao established the Department of Biology with two majors: biochemistry and biophysics. To solve the problem of faculty shortage, Prof. Tao arranged young teachers from Peking University, Sun Yat-sen University, and Shandong University to give courses in Jilin University. She also invited teachers from Jilin Normal University (now Northeast Normal University) to work as adjunct teachers. In September 1962, due to the economic difficulties in our country, the Ministry of Education decided to remove the Department of Biology in Jilin University. Upon Prof. Tao’s request, the major of biochemistry was kept and transferred to the Department of Chemistry, making it the first chemistry department with biochemistry as a major in China. During 1950s and 1960s, Prof. Tao focused her researches on plants in the northeast regions and carried out researches on sunflower seeds, cottonseeds, pumpkin seeds, red beans, soybeans, and other plant proteins, and published more than ten research articles. During the “Cultural Revolution”, Prof. Tao’s researches were suspended. And she was forced to go to Shulan in Jilin province in December 1969. She came back to Jilin University in December 1972 and immediately devoted herself into the study of the structure and function of nitrogenase. Together with her colleagues, Prof. Tao made great progresses on this study and published a series of research articles.

When the “Cultural Revolution” ended, Prof. Tao at the age of 80 years old was still full of enthusiasm in education. She encouraged the young teachers to go for advanced studies and trainings in high-level research universities and institutions worldwide to increase the quality of the faculty. Prof. Tao also helped the young teachers with their foreign language studies, guided them to choose their research focuses and encouraged them to write articles and compile textbooks. *The Molecular Basis of Protein*, a textbook about protein chemistry, was published by Higher Education Press in June 1981 and was widely used by peers within the discipline. Prof. Tao played an active role in academic activity. In 1979 and 1981, she participated in National Biochemistry Conference held in Hangzhou and Nanning respectively, and was elected as honorary director in the first and second conferences.

On December 11th, 1982, Prof. Tao passed away at the age of 87 in Changchun, Jilin province. According to her will, her husband Prof. Shizhi Guan donated their savings of 24,000 RMB to Jilin University for establishing *the Weisun Tao Scholarship* to sponsor the distinguished undergraduates in the Department of Chemistry.

**Figure 3:**
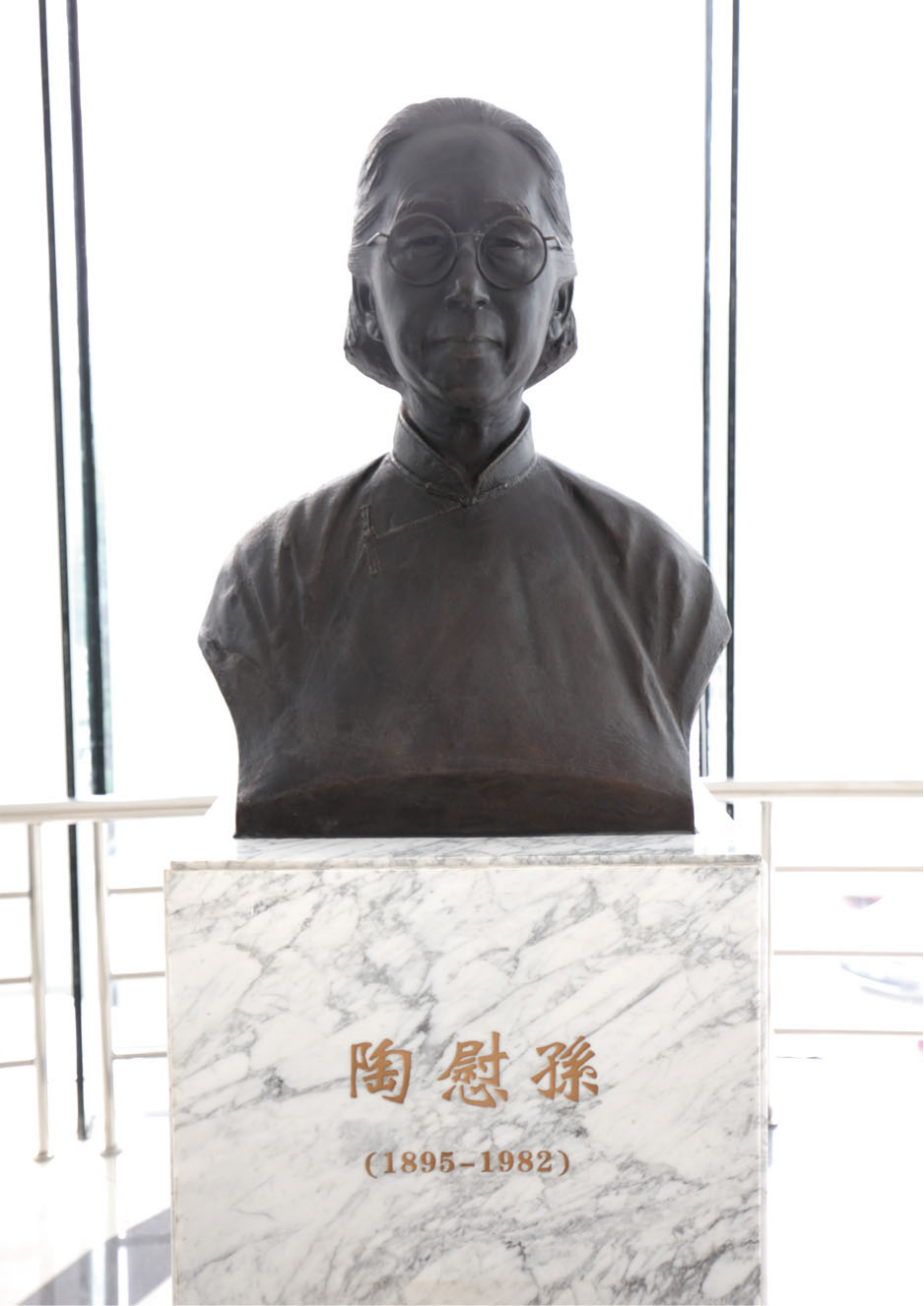
Bust of Professor Weisun Tao at Jilin University

After several generations of hard work, the chemistry discipline in Jilin University has now become Level I national key discipline, the biochemistry and molecular biology discipline in Jilin University has now become Level II national key discipline. In August 2012, commemorative activities were held for the 60th anniversary for the Department of Chemistry in Jilin University. The busts for Profs. Liusheng Tsai, Auchin Tang, Shizhi Guan, and Weisun Tao were built on campus in honor of their tremendous contributions to the university (Fig. 3).
